# Supplementary material for: Burnout and organisational stressors among healthcare staff working with adults with intellectual disabilities in Ireland
Source: PLoS One. 2025 Jan 28;20(1):e0313767. doi: 10.1371/journal.pone.0313767 (PMC11774381; doi:10.1371/journal.pone.0313767)
Supplement: S1 Table — Descriptive statistics for socio-demographic characteristics. (DOCX) [file pone.0313767.s001.docx]

**Burnout and organisational stressors among healthcare staff working with adults with intellectual disabilities in Ireland**

Patrick Clancy^1^* and Dr. Marica Cassarino^1^

1 School of Applied Psychology, University College of Cork, North Mall, Cork City, Ireland.

**S1 Table: Sample characteristics**

| *Sample characteristics* |  |  |
| --- | --- | --- |
|  | *n* | *%* |
| Gender (n=371) |  |  |
| Male | 32 | 8.6 |
| Female | 336 | 90.6 |
| Non- binary | 1 | 0.3 |
| Prefer not to say | 2 | 0.5 |
| Age (n=371) |  |  |
| 18- 29 | 89 | 24 |
| 30- 39 | 137 | 36.9 |
| 40- 49 | 89 | 24 |
| 50- 59 | 51 | 13.7 |
| 60- 69 | 5 | 1.3 |
| Duration of employment (n=371) |  |  |
| Less than one year | 24 | 6.5 |
| 1- 2 years | 54 | 14.6 |
| 3- 5 years | 119 | 32.1 |
| 6- 10 years | 79 | 21.3 |
| More than 10 years | 95 | 25.6 |
| Work- setting (n=371) |  |  |
| Day- service | 86 | 23.2 |
| Residential service | 214 | 57.7 |
| Both day and residential service | 54 | 14 |
| Other | 19 | 5.1 |
| Level of Education (n=371) |  |  |
| Secondary School | 18 | 4.9 |
| Undergraduate | 192 | 51.8 |
| Postgraduate | 161 | 43.4 |
| Hours worked in a week (n=371) |  |  |
| Less than 10 hours | 1 | 0.3 |
| 10- 19 hours | 9 | 2.4 |
| 20- 34 hours | 74 | 19.9 |
| 35- 40 hours | 184 | 49.6 |
| Over 40 hours | 103 | 27.8 |
| Covid- 19 pandemic impact on stress (n=303) |  |  |
| Worse or much worse | 253 | 83.5 |
| Same as before | 36 | 11.9 |
| Better or much better than before | 14 | 4.6 |
| Worked with service users with intellectual disabilities and mental health issues (n=324) |  |  |
| Never or rarely | 15 | 4.6 |
| Sometimes | 39 | 12 |
| Often | 89 | 27.5 |
| All the time | 181 | 55.9 |
