# Supplementary material for: Burnout and organisational stressors among healthcare staff working with adults with intellectual disabilities in Ireland
Source: PLoS One. 2025 Jan 28;20(1):e0313767. doi: 10.1371/journal.pone.0313767 (PMC11774381; doi:10.1371/journal.pone.0313767)
Supplement: S2 Table — Descriptive statistics for the Stress scales. (DOCX) [file pone.0313767.s002.docx]

**Burnout and organisational stressors among healthcare staff working with adults with intellectual disabilities in Ireland**

Patrick Clancy^1^* and Dr. Marica Cassarino^1^

1 School of Applied Psychology, University College of Cork, North Mall, Cork City, Ireland.

**S2 Table: SSQ Staff Stressor Questionnaire subscales**

|  | | | |
| --- | --- | --- | --- |
| *SSQ Staff Stressor Questionnaire Subscales* | | | |
|  | *n* | *M* | *SD* |
| Lack of Resources | 329 | 3.65 | 0.77 |
| Client challenging behaviour | 329 | 2.92 | 0.93 |
| Lack of staff support | 329 | 3.31 | 1.12 |
| Bureaucracy | 329 | 3.19 | 0.93 |
| Poor client skill | 329 | 2.37 | 0.81 |
| Low status job | 329 | 2.72 | 0.89 |
| Work-home conflict | 329 | 3.04 | 0.94 |
