# Supplementary material for: Burnout and organisational stressors among healthcare staff working with adults with intellectual disabilities in Ireland
Source: PLoS One. 2025 Jan 28;20(1):e0313767. doi: 10.1371/journal.pone.0313767 (PMC11774381; doi:10.1371/journal.pone.0313767)
Supplement: S3 Table — Bivariate correlations between measures. (DOCX) [file pone.0313767.s003.docx]

**Burnout and organisational stressors among healthcare staff working with adults with intellectual disabilities in Ireland**

Patrick Clancy^1^* and Dr. Marica Cassarino^1^

1 School of Applied Psychology, University College of Cork, North Mall, Cork City, Ireland.

**S3 Table: Pairwise Pearson’s correlations**

| *Pearson’s Correlations: CBI personal burnout, work- related burnout, client- related burnout, and the SSQ subscales* | | | | | | | | | |
| --- | --- | --- | --- | --- | --- | --- | --- | --- | --- |
| Measure | 2. Work- r. b. | 3. Clt- r. b. | 4. Lk of r. | 5. Cl. c. b. | 6. Pr c. sk. | 7. Lk of s. | 8. Low. s. j. | 9. Bure. | 10. W.- h. c. |
| 1. Personal burnout | 0.8*** | 0.43*** | 0.51*** | 0.38*** | 0.28*** | 0.38*** | 0.37*** | 0.43*** | 0.43*** |
| 2. Work- related b. |  | 0.57*** | 0.58*** | 0.45*** | 0.33*** | 0.45*** | 0.45*** | 0.48*** | 0.5*** |
| 3. Client- related b. |  |  | 0.34*** | 0.48*** | 0.45*** | 0.26*** | 0.34*** | 0.35*** | 0.32*** |
| 4. Lack of resources |  |  |  | 0.39*** | 0.3*** | 0.43*** | 0.33*** | 0.47*** | 0.42*** |
| 5. Client chall. beh. |  |  |  |  | 0.55*** | 0.43*** | 0.58*** | 0.39*** | 0.53*** |
| 6. Poor client skill |  |  |  |  |  | 0.23*** | 0.44*** | 0.41*** | 0.39*** |
| 7. Lack of staff sup. |  |  |  |  |  |  | 0.58*** | 0.41*** | 0.48*** |
| 8. Low job status |  |  |  |  |  |  |  | 0.42*** | 0.53*** |
| 9. Bureaucracy |  |  |  |  |  |  |  |  | 0.5*** |
| Notes: Statistical significance is presented as * p < .05, ** p < .01, *** p < .001  2. Work- r. b. = CBI Work- related burnout, 3. Clt- r. b. = CBI Client- related burnout, 4. Lk of r. = SSQ Lack of resources, 5. Cl. c. b. = SSQ Client- challenging behaviour, 6. Pr. c. sk. = SSQ Poor client skill, 7. Lk. of s. = SSQ Lack of staff support, 8. Low s. j. = SSQ Low- status job, 9. Bure. = SSQ Bureaucracy, 10. W.- h. c. = SSQ Work- home conflict | | | | | | | | | |
